# Severe and common mental disorders and risk of emergency hospital admissions for ambulatory care sensitive conditions among the UK Biobank cohort

**DOI:** 10.1192/bjo.2023.602

**Published:** 2023-11-07

**Authors:** Claire L. Niedzwiedz, María José Aragón, Josefien J. F. Breedvelt, Daniel J. Smith, Stephanie L. Prady, Rowena Jacobs

**Affiliations:** School of Health and Wellbeing, University of Glasgow, UK; Centre for Health Economics, University of York, UK; Centre for Urban Mental Health, University of Amsterdam, The Netherlands; School of Health and Wellbeing, University of Glasgow, UK; and Division of Psychiatry, Centre for Clinical Brain Sciences, University of Edinburgh, UK; Department of Health Sciences, University of York, UK

**Keywords:** Schizophrenia, bipolar type 1 or 2 disorders, anxiety or fear-related disorders, depressive disorders, ambulatory care sensitive conditions

## Abstract

**Background:**

People with mental disorders have worse physical health compared with the general population, which could be attributable to receiving poorer quality healthcare.

**Aims:**

To examine the relationship between severe and common mental disorders and risk of emergency hospital admissions for ambulatory care sensitive conditions (ACSCs), and factors associated with increased risk.

**Method:**

Baseline data for England (*N* = 445 814) were taken from UK Biobank, which recruited participants aged 37–73 years during 2006–2010, and linked to hospital admission records up to 31 December 2019. Participants were grouped into those with a history of either schizophrenia, bipolar disorder, depression or anxiety, or no mental disorder. Survival analysis was used to assess the risk of hospital admission for ACSCs among those with mental disorders compared with those without, adjusting for factors in different domains (sociodemographic, socioeconomic, health and biomarkers, health-related behaviours, social isolation and psychological).

**Results:**

People with schizophrenia had the highest (unadjusted) risk of hospital admission for ACSCs compared with those with no mental disorder (hazard ratio 4.40, 95% CI 4.04–4.80). People with bipolar disorder (hazard ratio 2.48, 95% CI 2.28–2.69) and depression or anxiety (hazard ratio 1.76, 95% CI 1.73–1.80) also had higher risk. Associations were more conservative when including all admissions, as opposed to first admissions only. The observed associations persisted after adjusting for a range of factors.

**Conclusions:**

People with severe mental disorders have the highest risk of preventable hospital admissions. Ensuring people with mental disorders receive adequate ambulatory care is essential to reduce the large health inequalities they experience.

People with mental disorders have double the risk of mortality compared with the general population, with a decade of years of potential life lost.^1^ The burden of mortality is highest among people with severe mental illness (SMI), including schizophrenia, bipolar disorder and other psychotic conditions, but is also elevated among those with common mental disorders (CMDs) such as depression and anxiety.^2^ Several studies have demonstrated that this excess mortality is mostly attributable to a higher burden of non-communicable diseases including cardiovascular disease, smoking-related lung disease and type 2 diabetes.^3–5^ Potential explanations for this include poorer quality of health and social care, lower adherence to treatment for physical health conditions, side-effects of psychotropic medications, unhealthier behaviours (e.g. smoking, alcohol consumption and physical inactivity) and underlying social inequalities.^5,6^

Hospital admissions for chronic illness represent a major proportion of overall healthcare spending.^7^ Therefore, preventing hospital admissions is likely to yield economic benefits, as well as reduce the overall burden on the health service. Ambulatory care sensitive conditions (ACSCs) are health conditions that are not considered to require in-patient treatment with appropriate management via primary care intervention.^8^ In England, ACSCs represent a sixth of emergency admissions, with an annual cost of £1.42 billion to the National Health Service (NHS).^9^ They therefore represent a key target for reduction, especially given the increasing trend over recent years.^10^ ACSCs can be grouped into acute (e.g. dehydration or gastroenteritis, where more severe progression can be prevented via early intervention), chronic (e.g. asthma, where effective care can reduce exacerbation of disease symptoms) or preventable (via vaccines and other interventions, e.g. influenza or pneumonia).^9^ Elevated levels of hospital admissions for ACSCs can be an indicator of poor continuity of care between primary and secondary care.^11^

Few studies have investigated hospital admissions for ACSCs among people with mental disorders, especially in the UK. Previous research from Denmark and Taiwan has demonstrated that people with SMI have higher risk of ACSC admissions, compared with those without.^6,12^ A study based in New York, USA, limited by its cross-sectional design and restricted geographic coverage, found that people with mental disorders were two times more likely to be admitted to hospital with an ACSC compared with those without a mental disorder.^13^ This was similar to a study based on the population of Western Australia, which used linked data from 1990 to 2006, and found that mental health patients were two times more likely to experience potentially preventable hospital admissions.^14^ Limitations of previous research include a lack of comparison between different mental disorders, with studies often either grouping all conditions together or focusing on one specific condition only.^12,15^ Research has also been limited by the sole use of electronic health records,^6,14,15^ which often do not contain sufficient data to investigate a range of potential covariates, such as income and social support. Most previous studies have also not taken into account the full burden of hospital admissions over time, often being restricted to the first admission or readmission within a certain time period.

## Objectives

Our objectives are to (a) examine the risk of emergency hospital admissions for ACSCs among individuals with and without severe and common mental disorders (SCMDs) (i.e. schizophrenia, bipolar disorder, depression and anxiety), using data from UK Biobank; and (b) explore the factors (sociodemographic, socioeconomic, health and biomarkers, health-related behaviours, social isolation and psychological factors) associated with any increased risk of ACSC admissions among people with SCMDs. Knowing the level of risk of ACSCs for people with SCMDs may help health services address key risk groups and risk factors, as well as implement preventive measures to reduce unnecessary healthcare utilisation.

## Method

### Data

For this cohort study, secondary data were taken from UK Biobank (https://www.ukbiobank.ac.uk/), which achieved a 5.5% response rate.^16,17^ Over 502 000 community-dwelling individuals aged 37–73 years were recruited to UK Biobank during 2006–2010. Participants attended one of 22 assessment centres across England, Scotland and Wales. For this study, we limited the sample to those attending assessment centres in England. Baseline assessments were linked to Hospital Episode Statistics (HES) for England and death records provided by NHS Digital (both up to 31 December 2019). The authors assert that all procedures contributing to this work comply with the ethical standards of the relevant national and institutional committees on human experimentation and with the Helsinki Declaration of 1975, as revised in 2008. UK Biobank was approved by NHS National Research Ethics Service North West (approval number 21/NW/0157). All adult participants provided written informed consent to participate in UK Biobank. We excluded participants who requested their data be withdrawn from UK Biobank (updated on 9 August 2021).

### Cohort definition

Individuals with an SCMD were identified via linked clinical records and/or self-report, using the ‘first occurrence’ variables (see https://biobank.ndph.ox.ac.uk/ukb/label.cgi?id=1712 for full details). UK Biobank provides the date on which a diagnosis was recorded for the first time and the source (e.g. primary care, in-patient data or self-reported data). For each diagnosis group of interest (bipolar disorder (ICD-10 codes F30, F31), schizophrenia and other psychotic disorders (ICD-10 codes F20–F29), depression (ICD-10 codes F32–F39) and anxiety and related disorders (ICD-10 codes F40–F48)), the earliest date on which a diagnosis was recorded (from linked primary care or hospital in-patient data) was identified.

At the baseline assessment centre individuals could also self-report a lifetime diagnosis of ‘schizophrenia’; ‘mania/bipolar disorder/manic depression’; ‘depression’ or anxiety and related disorders: ‘anxiety/panic attacks’, ‘nervous breakdown’, ‘post-traumatic stress disorder’, ‘obsessive–compulsive disorder’ or ‘stress’. A subset of UK Biobank participants (those recruited in 2009–2010) also completed detailed questionnaires about lifetime depressive and mania symptoms at the baseline assessment, from which probable cases of major depression and bipolar disorder have been derived by clinicians.^18^ For solely self-reported records, the date of diagnosis was recorded as the date the individual joined UK Biobank. An individual was classified as having the corresponding mental disorder diagnosis if they had either a clinical record and/or a self-reported record.

If a person had more than one SCMD diagnosis, we ranked them in the following order and classified the patient according to the highest ranked: schizophrenia, bipolar disorder, anxiety or depression. Participants who had an ICD-10 code under Chapter V (mental and behavioural disorders (e.g. eating disorders or as a result of psychoactive substance use)) not covered by the mental disorder categories above, were excluded from the sample (*n* = 31 923), and all those with no recorded mental or behavioural disorder were grouped into the control group. For the analysis, we grouped those with anxiety or depression (CMDs) together because of their significant comorbidity.^19^

### Outcome

The primary outcome of interest was emergency hospital admissions for an ACSC. ACSCs were defined according to the NHS England criteria, which includes 19 conditions divided into acute, chronic and vaccine-preventable.^20^ Acute conditions included cellulitis; dental conditions; ear, nose and throat infections; gangrene; gastroenteritis/dehydration; nutritional deficiency; pelvic inflammatory disease; perforated/bleeding ulcers and pyelonephritis. Chronic conditions included angina, asthma, chronic obstructive pulmonary disease, congestive heart failure, diabetes complications, convulsions/epilepsy, hypertension and iron deficiency anaemia. Vaccine-preventable conditions included influenza and pneumonia and ‘others’ such as tuberculosis and hepatitis B.

Hospital admissions for ACSCs were extracted from the HES for England supplied by NHS Digital via UK Biobank.^21^ HES reports data as episodes (period of care under a consultant), and there can be more than one episode during one hospital stay. Each episode can record multiple diagnoses (via ICD-10 codes), which can be used to identify ACSCs. An ACSC admission was defined as an ACSC condition recorded in the first emergency admission episode.

To identify ACSC admissions, we grouped together consecutive episodes of the same patient. UK Biobank does not report hospital codes, so these continuous periods in hospital can include transfers between different hospitals. To construct these ‘continuous in-patient spells’, we used information about the source and method of admission and the discharge destination, together with the start and end dates of the episodes to make sure the episodes were in the correct order. Supplementary Table 1 available at https://doi.org/10.1192/bjo.2023.602 contains detail on the data exclusions in HES to identify ACSC admissions.

### Covariates

We included a range of potential variables that may influence the association between SCMD and ACSC admissions, grouped into sociodemographic, socioeconomic, health and biomarkers, health-related behaviours, social isolation and psychological factors. All data for the covariates were collected at the baseline assessment centre.

Sociodemographic factors included age (years), gender (male, female), ethnicity (White British, White Irish, other White background, South Asian, Black, mixed or other), urban/rural residence (based on home postcode population density) and assessment centre attended. Socioeconomic factors included education level (1: university or college degree; 2: A-levels or equivalent; 3: O-levels, General Certificate of Secondary Education (GCSE), vocational Certificate of Secondary Education (CSE) or equivalent; 4: other (e.g. National Vocational Qualifications or other professional qualifications) or 5: none of the above), deprivation at the output area level (assessed with the Townsend index,^22^ converted to a *Z*-score (number of s.d.s from the mean value) where higher levels reflect higher levels of deprivation), employment status (paid employment or self-employment, retired, looking after home and/or family, unable to work because of sickness or disability, unemployment or other), housing tenure (owner-occupier or renter/other) and household income (before tax, self-reported: <£18 000, £18 000–£30 999, £31 000–£51 999, £52 000–£100 000 or >£100 000).

Health measures included multimorbidity (a count of the number of self-reported chronic physical health conditions (0, 1, 2, 3 or ≥4), based on a previously published approach,^23^ excluding mental health conditions) and body mass index (BMI) category (underweight, normal weight, overweight, obese). We included three biomarkers indicative of inflammation (C-reactive protein (CRP), logged because of its skewed distribution), metabolic function (waist circumference) and cardiovascular function (pulse rate). Indicators of health behaviours included smoking (never, previous, current) and alcohol consumption (daily or almost daily, 3–4 times a week, once or twice a week, 1–3 times per month, special occasions, former drinker or never). Physical activity (walking, moderate and vigorous) in a typical week was also recorded with self-reported items from the International Physical Activity Questionnaire Short Form,^24^ from which a single measure of total physical activity in metabolic equivalent of task hours per week was derived; this was converted into quintiles.^25^

A number of measures were used to capture social isolation: living arrangements (with spouse/partner, with other people, live alone), social contact (visit friends/family less than weekly versus at least once a week) and social participation (one or more activity, e.g. sports club, at least once a week versus no activities). Finally, psychological factors included loneliness (whether participants often feel lonely, yes or no), current depressive symptoms (measured with an adapted Patient Health Questionnaire-4)^26^ and sleeplessness (never/rarely, sometimes, usually).

### Statistical analysis

First, descriptive statistics for the sample were calculated, including the number of hospital admissions by SCMD diagnosis. We then ran several survival models to assess the relationship between SCMD (schizophrenia, bipolar disorder, depression or anxiety, and those with no disorder as the reference group) and ACSC admissions. We ran models in the following order to examine the associations using different groups of covariates, in particular those over and above sociodemographic and socioeconomic factors that are most often included in previous studies:^6^

- unadjusted;
- model 1 plus age, gender, ethnicity, urban/rural and assessment centre location (sociodemographic factors);
- model 2 plus education, deprivation, employment status, housing tenure and income (socioeconomic factors);
- model 3 plus multimorbidity, BMI, pulse rate, waist circumference and CRP (health and biomarker variables);
- model 3 plus smoking, alcohol consumption and physical activity (health-related behaviours);
- model 3 plus living arrangements, social participation and social contact (social isolation factors);
- model 3 plus depressive symptoms, sleeplessness and loneliness (psychological factors);
- all variables.

The observation period for each person started on the date of the initial baseline UK Biobank assessment centre attendance or when they were diagnosed with an SCMD, if the diagnosis was later than the assessment. During the observation period, a patient could have none, one or more than one hospital admission, and we included participants who had ACSC admissions before joining UK Biobank, because of the older age of participants. Models were censored at the earliest date of ACSC admission, date of death or the end of follow-up on 31 December 2019. We use two model specifications, one modelling the time to first admission within the study period (Cox proportional hazard model) and one that considered all admissions (Prentice–Williams–Peterson total time (PWP-TT) model).^27,28^ The time to first admission model does not use all data (it ignores second and later admissions), and can show associations between covariates and admissions that do not hold once all admissions are considered.^27^ The PWP-TT analyses ordered multiple events via stratification, based on the prior number of events during the follow-up period.^28,29^ It therefore takes into account that having a prior admission affects the risk of future admissions, and that the effect of covariates may differ in subsequent events.^28^ Further details on these models and how they can be implemented in Stata software can be found elsewhere.^27^ To maximise the use of available data, participants with missing data for any variable were excluded from the analysis by using pairwise deletion (models contain a different number of individuals and therefore should not be directly compared). The extent of missing data varied from 1.5% in model 2 to 35.5% in model 8 (mainly because of the high proportion of missing data relating to physical activity). Violations of the proportional hazard assumption were examined graphically by plotting scaled Schoenfeld residuals. Statistical analysis was performed with software Stata/MP version 17 for Windows.

## Results

### Description of sample

**Fig. 1:**
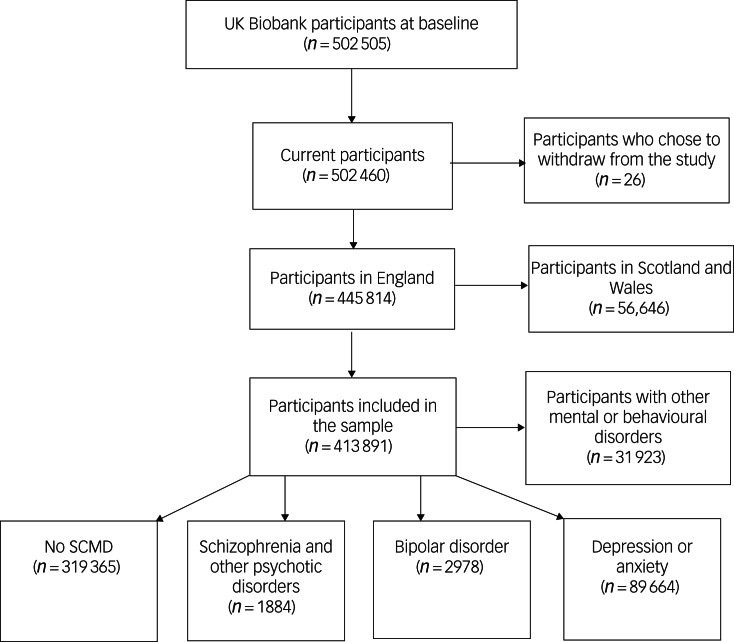
Flowchart of study participants. SCMD, severe and common mental disorder.

Our sample comprised 413 891 participants (Fig. 1) who attended an assessment centre in England and had either no prior psychiatric disorder diagnosis (*n* = 319 365) or a previous diagnosis of schizophrenia (*n* = 1884), bipolar disorder (*n* = 2978) or anxiety/depression (*n* = 89 664). Most participants with an SCMD experienced a hospital admission during the 13-year follow-up period, with over half experiencing an emergency admission; 10 832 participants experienced an emergency ACSC admission, with 7218 experiencing just one admission and 504 experiencing more than five (Supplementary Table 2). Table 1 (and Supplementary Table 3) shows the descriptive statistics (derived from the model containing all covariates, excluding missing data) by SCMD diagnosis. Across all diagnosis groups, individuals with an SCMD were less likely to be in paid employment and more likely to live in deprived areas, experience poorer overall health, have adverse health behaviours and be socially isolated, compared with those without an SCMD.

**Table 1:** Descriptive statistics for the sample (shown as proportions unless otherwise specified)

	No SCMD	Schizophrenia	Bipolar disorder	Depression or anxiety
Number of observations^a^	212 074	1114	2085	60 553
Number of ACSC admissions	16 656	370	450	9334
Number of participants	207 525	922	1878	56 637
Number of participants with ACSC admissions	12 107	178	243	5418
Number of participants who died during follow-up	8,117	152	140	3178
Sociodemographic variables				
Age, years, mean	56.39	55.34	55.53	55.86
Gender, female	0.50	0.43	0.52	0.62
Gender, male	0.50	0.57	0.48	0.38
Ethnicity, White British	0.89	0.82	0.85	0.90
Ethnicity, White Irish	0.03	0.04	0.04	0.03
Ethnicity, White other	0.03	0.04	0.05	0.03
Ethnicity, mixed	0.01	0.02	0.01	0.01
Ethnicity, South Asian	0.02	0.02	0.02	0.01
Ethnicity, Black	0.01	0.04	0.02	0.01
Ethnicity, other	0.01	0.02	0.01	0.01
Rural, urban	0.85	0.93	0.90	0.86
Rural, rural	0.15	0.07	0.10	0.14
Socioeconomic variables				
Education, college or University	0.38	0.30	0.40	0.34
Education, A/AS levels	0.12	0.16	0.13	0.12
Education, O levels/GCSE	0.26	0.24	0.25	0.28
Education, other	0.11	0.10	0.10	0.12
Education, none of the above	0.12	0.21	0.12	0.15
Townsend deprivation index, mean^b^	−1.62	1.25	−0.26	−1.08
Employment status, paid employment	0.62	0.26	0.48	0.56
Employment status, retired	0.32	0.32	0.30	0.31
Employment status, looking after home/family	0.02	0.03	0.03	0.03
Employment status, unable to work	0.01	0.32	0.14	0.06
Employment status, unemployed	0.01	0.04	0.03	0.02
Employment status, other	0.01	0.03	0.03	0.01
Housing, own	0.93	0.55	0.77	0.86
Housing, rent/other	0.07	0.45	0.23	0.14
Household income, <£18 000	0.18	0.66	0.37	0.29
Household income, £18 000–£30 999	0.25	0.19	0.24	0.26
Household income: £31 000–£51 999	0.27	0.09	0.21	0.24
Household income: £52 000–£100 000	0.23	0.05	0.14	0.17
Household income: >£100 000	0.07	0.02	0.03	0.04
Health and biomarkers				
BMI, underweight	0.00	0.02	0.01	0.01
BMI, normal	0.34	0.27	0.29	0.32
BMI, overweight	0.43	0.35	0.38	0.40
BMI, obese	0.22	0.36	0.32	0.27
Pulse rate, mean	68.86	75.85	70.66	70.07
Waist circumference, mean	89.97	96.20	93.75	90.67
C-reactive protein [log]^c^	0.24	0.67	0.47	0.41
Number of comorbidities,^d^ 0	0.40	0.14	0.20	0.29
Number of comorbidities, 1	0.34	0.28	0.29	0.32
Number of comorbidities, 2	0.17	0.32	0.25	0.21
Number of comorbidities, 3	0.07	0.14	0.14	0.11
Number of comorbidities, ≥4	0.03	0.12	0.12	0.08
Health-related behaviours				
Smoking, never	0.59	0.43	0.43	0.49
Smoking, previous	0.35	0.29	0.35	0.37
Smoking, current	0.06	0.29	0.22	0.14
Alcohol, daily or almost daily	0.22	0.17	0.22	0.20
Alcohol, 3–4 times/week	0.26	0.14	0.17	0.21
Alcohol, 1–2 times/week	0.26	0.19	0.22	0.24
Alcohol, 1–3 times/month	0.11	0.11	0.12	0.12
Alcohol, special occasions only	0.10	0.16	0.13	0.13
Alcohol, never (former drinker)	0.03	0.18	0.08	0.05
Alcohol, never	0.04	0.06	0.05	0.04
Physical activity quintile 1	0.19	0.31	0.22	0.22
Physical activity quintile 2	0.20	0.22	0.21	0.20
Physical activity quintile 3	0.20	0.18	0.19	0.19
Physical activity quintile 4	0.21	0.14	0.19	0.19
Physical activity quintile 5	0.20	0.16	0.20	0.20
Social isolation				
Household structure, live with spouse/partner	0.77	0.35	0.58	0.65
Household structure, live with other person	0.07	0.10	0.13	0.11
Household structure, live alone	0.17	0.55	0.29	0.24
Visits friends/family once or more per week	0.78	0.70	0.75	0.78
Visits friends/family less than once per week	0.22	0.30	0.25	0.22
Leisure/social activities once or more per week	0.72	0.69	0.71	0.68
Leisure/social activities less than once per week	0.28	0.31	0.29	0.32
Psychological factors				
Lonely, no	0.87	0.60	0.59	0.69
Lonely, yes	0.13	0.40	0.41	0.31
PHQ, mean	1.23	3.59	3.42	2.61
Insomnia, never/rarely	0.28	0.20	0.19	0.18
Insomnia, sometimes	0.48	0.48	0.44	0.45
Insomnia, usually	0.24	0.32	0.37	0.36

SCMD, severe and common mental disorder; ACSC, ambulatory care sensitive condition; BMI, body mass index; PHQ, Patient Health Questionnaire.

a. Includes participants who may have had more than one relevant hospital admission and so are counted more than once.

b. *Z*-score (higher values reflect greater deprivation).

c. Logged owing to skewed distribution.

d. Count of the number of self-reported chronic physical health conditions.

### Risk of ACSC hospital admissions

When looking at the first admission only in unadjusted models, people with schizophrenia had the highest risk of ACSC admission compared with those with no mental disorder (hazard ratio 4.40, 95% CI 4.04–4.80) (Table 2). People with bipolar disorder (hazard ratio 2.48, 95% CI 2.28–2.69) and depression or anxiety (hazard ratio 1.76, 95% CI 1.73–1.80) also had heightened risk. When taking multiple admissions into account (Table 3), the associations were weaker but still elevated (schizophrenia: hazard ratio 2.29, 95% CI 2.08–2.52; bipolar disorder: hazard ratio 1.92, 95% CI 1.78–2.08; depression or anxiety: hazard ratio 1.57, 95% CI 1.53–1.60).

**Table 2:** Results from Cox proportional hazard models for the association between severe and common mental disorders and risk of hospital admission for ambulatory care sensitive conditions (first admission per person only)

	Model 1	Model 2	Model 3	Model 4	Model 5	Model 6	Model 7	Model 8
	Hazard ratio [95% CI]	Hazard ratio [95% CI]	Hazard ratio [95% CI]	Hazard ratio [95% CI]	Hazard ratio [95% CI]	Hazard ratio [95% CI]	Hazard ratio [95% CI]	Hazard ratio [95% CI]
Reference: no SCMD diagnosis								
Schizophrenia	4.40	4.86	2.65	2.31	2.47	2.65	2.56	2.15
	[4.04–4.80]	[4.44–5.32]	[2.37–2.97]	[2.05–2.61]	[2.19–2.79]	[2.36–2.97]	[2.26–2.89]	[1.87–2.48]
Bipolar disorder	2.48	2.81	2.12	1.72	1.95	2.10	1.95	1.56
	[2.28–2.69]	[2.58–3.06]	[1.92–2.33]	[1.55–1.90]	[1.76–2.16]	[1.90–2.31]	[1.76–2.16]	[1.39–1.76]
Anxiety or depression	1.76	1.98	1.72	1.53	1.63	1.71	1.59	1.43
	[1.73–1.80]	[1.93–2.02]	[1.68–1.77]	[1.49–1.57]	[1.59–1.67]	[1.67–1.75]	[1.55–1.64]	[1.39–1.47]
Covariates included								
Sociodemographic	No	Yes	Yes	Yes	Yes	Yes	Yes	Yes
Socioeconomic	No	No	Yes	Yes	Yes	Yes	Yes	Yes
Health and biomarkers	No	No	No	Yes	No	No	No	Yes
Health-related behaviours	No	No	No	No	Yes	No	No	Yes
Social isolation	No	No	No	No	No	Yes	No	Yes
Psychological	No	No	No	No	No	No	Yes	Yes
Number of observations	413 203	407 028	342 143	313 770	319 173	340 337	309 100	266 616
Number of participants	413 203	407 028	342 143	313 770	319 173	340 337	309 100	266 616
Number of ACSC admissions	44 802	44 164	35 412	31 984	31 713	35 177	31 343	25 818

SCMD, severe and common mental disorder; ACSC, ambulatory care sensitive condition.

**Table 3:** Results from Prentice–Williams–Peterson total time models for the association between severe and common mental disorders and risk of hospital admissions for ambulatory care sensitive conditions (all admissions)

	Model 1	Model 2	Model 3	Model 4	Model 5	Model 6	Model 7	Model 8
	Hazard ratio [95% CI]	Hazard ratio [95% CI]	Hazard ratio [95% CI]	Hazard ratio [95% CI]	Hazard ratio [95% CI]	Hazard ratio [95% CI]	Hazard ratio [95% CI]	Hazard ratio [95% CI]
Reference: no SCMD diagnosis								
Schizophrenia	2.29	2.64	2.09	1.96	1.92	2.10	2.11	1.86
	[2.08–2.52]	[2.38–2.93]	[1.84–2.38]	[1.70–2.25]	[1.66–2.23]	[1.85–2.39]	[1.83–2.42]	[1.57–2.20]
Bipolar disorder	1.92	2.13	1.79	1.58	1.84	1.78	1.73	1.62
	[1.77–2.08]	[1.95–2.33]	[1.62–1.98]	[1.42–1.76]	[1.68–2.03]	[1.61–1.97]	[1.55–1.92]	[1.46–1.80]
Anxiety or depression	1.57	1.69	1.54	1.42	1.48	1.53	1.47	1.36
	[1.54–1.60]	[1.66–1.73]	[1.50–1.58]	[1.38–1.46]	[1.44–1.52]	[1.49–1.57]	[1.43–1.51]	[1.32–1.40]
Covariates included								
Sociodemographic	No	Yes	Yes	Yes	Yes	Yes	Yes	Yes
Socioeconomic	No	No	Yes	Yes	Yes	Yes	Yes	Yes
Health and biomarkers	No	No	No	Yes	No	No	No	Yes
Health-related behaviours	No	No	No	No	Yes	No	No	Yes
Social isolation	No	No	No	No	No	Yes	No	Yes
Psychological	No	No	No	No	No	No	Yes	Yes
Number of observations	432 063	425 577	356 098	325 908	330 593	354 161	321 175	275 476
Number of participants	413 504	407 321	342 359	313 954	319 356	340 553	309 283	266 751
Number of ACSC admissions	63 662	62 713	49 367	44 122	43 133	49 001	43 418	34 678

SCMD, severe and common mental disorder; ACSC, ambulatory care sensitive condition.

**Fig. 2:**
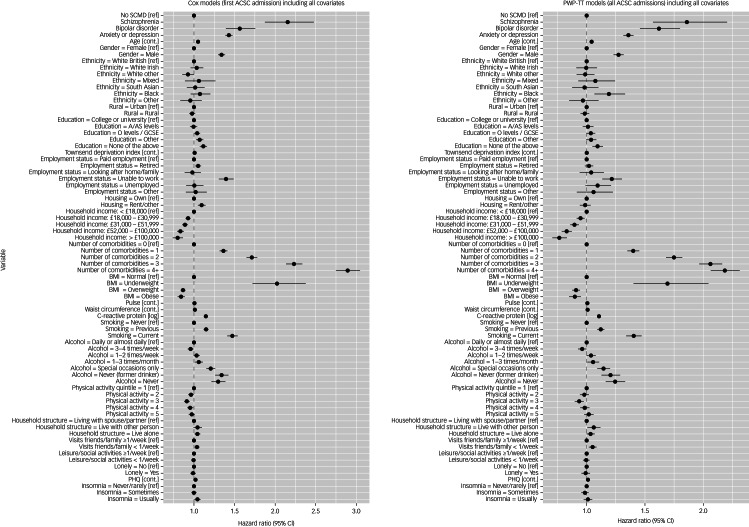
Results from models for the association between SCMD and emergency hospital admissions for ACSCs, including all variables. SCMD, severe and common mental disorder; ACSC, ambulatory care sensitive condition; BMI, body mass index; cont., continuous variable; PHQ, Patient Health Questionnaire; PWP-TT, Prentice–Williams–Peterson total time; ref, reference category.

With the addition of socioeconomic factors, associations remained positive (Table 2 and Supplementary Table 4), with the strongest association observed for those with schizophrenia (hazard ratio 2.65, 95% CI 2.37–2.97), in models examining the first admission. The associations were attenuated with the addition of health and biomarker variables. Inclusion of the social isolation variables and the psychological variables did not alter associations. The pattern of results was generally similar when all admissions were considered, with all mental health conditions showing increased risk of ACSC admissions, but with less attenuation following inclusion of different covariates (Table 3, Fig. 2 and Supplementary Table 5).

### Risk of hospital admissions associated with different covariates

Having a previous diagnosis of a severe mental disorder was one of the strongest predictors of hospital admission for ACSCs (Fig. 2). Of the sociodemographic factors examined (model 2), increased age, being male and belonging to an ethnic minority group (apart from the White other category) were associated with increased risk of ACSC admission (Supplementary Table 4). Those living in a rural area were less likely to experience an ACSC admission compared with those from an urban area. Having fewer educational qualifications, not being in employment and not being a home owner were all associated with increased risk of ACSC admissions (model 3). Similarly, there was a gradient in the risk of ACSC admission according to household income, with those earning >£100 000 per year showing the lowest risk of ACSC admission, compared with those earning <£18 000. One of the strongest associations observed among the covariates included was when multimorbidity was added to the model. People with four or more physical comorbidities had a threefold higher risk of ACSC admission compared with those without any comorbidity (model 4). Higher levels of CRP were also related to increased risk of ACSC admissions in model 4. Current smokers, and to a lesser extent previous smokers, had higher risk of ACSC admissions compared with never smokers (model 5). Loneliness was not related to risk of ACSC admission, whereas experiencing higher levels of depressive symptoms and insomnia were related to increased risk (model 7). In the model containing all predictors (model 8), an SCMD diagnosis was still associated with elevated risk of ACSC admission and most other factors also remained associated. Findings were relatively similar when taking all ACSC admissions into account (Supplementary Table 5).

## Discussion

### Summary of findings

In our study of UK Biobank participants, we found that a previous diagnosis of schizophrenia (or other psychotic disorder) was one of the strongest predictors of potentially preventable hospital admissions. Bipolar disorder and anxiety/depression were also strongly associated. Adjustment for socioeconomic circumstances reduced the associations observed, but they persisted even when accounting for a number of different variables, such as biomarkers, health-related behaviours, social isolation and psychological factors. Including health and biomarker variables attenuated the association between SCMD and ACSC admissions, but the inclusion of social isolation and psychological factors (including loneliness) made little difference. Models that considered all hospital admissions for ACSCs, as opposed to just the first event during the study period, displayed a similar pattern of results, although the associations were more conservative overall.

Our findings are in line with the few studies from other countries that demonstrate elevated risk of ACSC-related hospital admissions among people with severe mental disorders.^6^ Less research has focused on CMDs, with most only looking at depression.^15^ A prior study found elevated levels of ACSC admissions among those with anxiety, but this was among a cohort of veterans with diabetes, who were not likely to be representative of the wider population.^30^ Our study extends this research by including a wide range of covariates that have not been considered in previous studies, and by examining all ACSC admissions across a 13-year period. Estimates derived from the models including all ACSC admissions during the study period were more conservative compared with those including only the first admission. This is consistent with previous research accounting for multiple admissions, as the underlying risk of hospital admission increases as the number of accumulated admissions increases.^27,31^ The finding that socioeconomic variables appear to make a key contribution to risk of ACSC admissions among those with SCMDs suggests that more needs to be done to reduce socioeconomic inequalities experienced by those with mental disorders, and in particular people with schizophrenia and other psychotic disorders.

### Strengths and limitations

A key strength of our study was the use of UK Biobank data, which enabled the exploration of a range of different variables that may influence ACSC admissions. Administrative data alone often lacks detail on important socioeconomic, psychological and health risk variables,^6,13^ but UK Biobank enables the linkage of these variables to administrative health records. The large sample size and broad phenotyping provided by UK Biobank also allowed us to examine more detailed psychiatric diagnoses than has been conducted previously, with most prior research combining schizophrenia and bipolar diagnostic groups and not including a comparison to those with CMDs, or focusing on depression in isolation.^6,15^ Another significant strength of our analysis was the investigation of multiple hospital admissions per person over a long 13-year follow-up time.

However, a key limitation is that UK Biobank is not representative of the general UK population, with White, more advantaged and healthier people being more likely to participate.^32^ Selection bias therefore limits the internal and external validity of the results. This is potentially important for individuals with SMI, as those with more serious illness, who may also have more physical health issues, may be less likely to participate.^33^ Consequences of this may include underestimation of the associations observed.^34^ UK Biobank is also susceptible to survival bias, as most people were aged 40–70 years at recruitment, and we know from previous research that those with SMI are more likely to die prematurely.^2^ The degree of missing data in our analyses (particularly high for physical activity) may have introduced additional bias, with the assumption that they were missing completely at random unlikely to be met. Similarly, comparison between our models is limited because of the different samples within each model as a result of missing data. Further research that explores patterns of non-participation and missing data among those with SCMDs within non-representative samples, such as provided by UK Biobank, is needed to unpack the effect on associations with physical health outcomes.

In conclusion, people with severe mental disorders had the highest risk of preventable hospital admissions, with the risk also elevated among individuals with depression and anxiety. This may represent an unmet need for high-quality community and primary preventive care. Lack of access to primary care has been noted to be more prevalent among those with mental illness,^35^ with preventive activities in primary care shown to reduce unplanned hospital admissions.^36^ Ensuring those with mental disorders (particularly SMI) receive adequate primary healthcare and targeting interventions at multiple levels, including the individual (e.g. smoking cessation, reducing loneliness/social isolation), health system (improved care coordination, outreach) and broader community/society (reduced unemployment, poverty and discrimination),^37,38^ may help to reduce the burden of potentially avoidable hospital admissions experienced by these groups. Some studies suggest that integrated care models can lead to improved medical outcomes for people with mental health problems.^39^ There are also opportunities for improved care following a hospital admission, to prevent repeat admissions.^40^ Policy implications of our findings to reduce potentially avoidable acute care use may include the need for greater integration of mental and physical healthcare, health and care professionals playing a role in taking a ‘whole person’ perspective toward the physical and mental health needs of people with SCMDs, ensuring equity of access for physical healthcare needs for people with SCMDs and targeting preventive interventions that have been shown to be effective in addressing physical health needs.

## Supporting information

Niedzwiedz et al. supplementary material Niedzwiedz et al. supplementary material

## Data Availability

The data/research material that support the findings of this study are available from UK Biobank (https://www.ukbiobank.ac.uk/), but restrictions apply to their availability. The data were used under licence for the current study and so are not publicly available. The data are available from the authors upon reasonable request and with permission of UK Biobank. Analytical code for the study is available from the authors on request.
