# Supplementary material for: Severe and common mental disorders and risk of emergency hospital admissions for ambulatory care sensitive conditions among the UK Biobank cohort
Source: BJPsych Open. 2023 Nov 7;9(6):e211. doi: 10.1192/bjo.2023.602 (PMC10753948; doi:10.1192/bjo.2023.602)
Supplement: Niedzwiedz et al. supplementary material [file S2056472423006026sup001.docx]

# Supplementary Material

Severe and common mental disorders and risk of emergency hospital admissions for Ambulatory Care Sensitive Conditions (ACSCs) among the UK Biobank cohort

**Authors**

Claire L. Niedzwiedz^1*^ (E-mail : claire.niedzwiedz@glasgow.ac.uk)

María José Aragón

Josefien J. F. Breedvelt

Daniel J. Smith

Stephanie L. Prady

Rowena Jacobs

^1^ School of Health & Wellbeing, University of Glasgow, Clarice Pears Building, 90 Byres Rd, Glasgow G12 8TB, UK

^*^ Corresponding author

**Contents:**

[Table S1: HES-APC data: Identify ACSC CIPS 2](#_Toc146807173)

[Table S2: Number of ACSC admissions among participants people with a severe or common mental disorder (SCMD) diagnosis 3](#_Toc146807174)

[Table S3: Descriptive statistics for the sample (proportions and 95% confidence intervals in brackets unless specified) 4](#_Toc146807175)

[Table S4: Full results from the Cox Proportional Hazard models (first admission) for the association between SCMD and ACSC admissions 8](#_Toc146807176)

[Table S5: Full results from the Prentice, Williams and Peterson Total Time (PWP-TT] models (all admissions) for the association between SCMD and ACSC admissions 12](#_Toc146807177)

Table S1: HES-APC data: Identify ACSC CIPS

|  | Number of Episodes / CIPS | Number of Patients | SCMD Episodes / CIPS | SCMD Patients |
| --- | --- | --- | --- | --- |
| HES Episodes | 3,341,300 | 392,049 | 952,194 | 87,862 |
| Excluded: |  |  |  |  |
| Unfinished episodes | 409 | 3 | 213 | 0 |
| No start date | 213 | 7 | 65 | 1 |
| No end date | 20 | 0 | 11 | 0 |
| Negative duration | 38 | 0 | 11 | 0 |
| Patients with MH, but non-SMCD diagnosis | 351,044 | 29,866 | 0 | 0 |
| Admission before patient joined Biobank | 924,753 | 44,124 | 305,699 | 8,452 |
| After 31 December 2019 | 236,425 | 10,428 | 67,401 | 1,810 |
| Admission before SMI diagnosis | 52,872 | 769 | 52,872 | 769 |
| Elective and maternity | 1,279,270 | 159,818 | 347,066 | 34,546 |
| Duplicates | 445 | 0 | 164 | 0 |
| Final Number of Episodes | 495,811 | 147,034 | 178,692 | 42,284 |
| CIPS | 330,932 | 147,034 | 116,691 | 42,284 |
| ACSC CIPS | 50,677 | 31,787 | 20,651 | 10,859 |

The table above starts with the total number of episodes and patients in HES, then it shows the patients that were excluded due to different reasons. The last three rows show the final number of episodes, after all exclusions, the number of CIPS these episodes form and how many of them are ACSC CIPS.

*Table S2: Number of ACSC admissions among participants people with a severe or common mental disorder (SCMD) diagnosis*

| **Number of Admissions** | **Total Participants** | **Participants with no SCMD** | **Participants with SCMD** |
| --- | --- | --- | --- |
| 0 | 382,220 | 298,526 | 83,694 |
| 1 | 23,192 | 15,974 | 7,218 |
| 2 | 4,769 | 3,010 | 1,759 |
| 3 | 1,691 | 913 | 778 |
| 4 | 818 | 447 | 371 |
| 5 | 416 | 214 | 202 |
| >5 | 785 | 281 | 504 |
| **Total** | **413,891** | **319,365** | **94,526** |

Table S3: Descriptive statistics for the sample (proportions and 95% confidence intervals in brackets unless specified)

|  | **No SCMD** | **Schizophrenia** | **Bipolar** | **Depression or Anxiety** |
| --- | --- | --- | --- | --- |
| Number of Observations | 212,074 | 1,114 | 2,085 | 60,553 |
| Number of ACSC admissions | 16,656 | 370 | 450 | 9,334 |
| Number of Patients | 207,525 | 922 | 1,878 | 56,637 |
| Number of Patients with ACSC admissions | 12,107 | 178 | 243 | 5,418 |
| Number of Patients who died | 8,117 | 152 | 140 | 3,178 |
| **Sociodemographic:** Age (mean) | 56.39 | 55.34 | 55.53 | 55.86 |
|  | [56.36 - 56.43] | [54.87 - 55.81] | [55.18 - 55.87] | [55.8 - 55.93] |
| Sex = Female | 0.50 | 0.43 | 0.52 | 0.62 |
|  | [0.49 - 0.5] | [0.4 - 0.46] | [0.49 - 0.54] | [0.62 - 0.62] |
| Sex = Male | 0.50 | 0.57 | 0.48 | 0.38 |
|  | [0.5 - 0.51] | [0.54 - 0.6] | [0.46 - 0.51] | [0.38 - 0.38] |
| Ethnicity = White British | 0.89 | 0.82 | 0.85 | 0.90 |
|  | [0.89 - 0.9] | [0.8 - 0.84] | [0.83 - 0.86] | [0.9 - 0.91] |
| Ethnicity = White Irish | 0.03 | 0.04 | 0.04 | 0.03 |
|  | [0.02 - 0.03] | [0.03 - 0.06] | [0.03 - 0.05] | [0.03 - 0.03] |
| Ethnicity = White Other | 0.03 | 0.04 | 0.05 | 0.03 |
|  | [0.03 - 0.03] | [0.03 - 0.05] | [0.04 - 0.06] | [0.03 - 0.03] |
| Ethnicity = Mixed | 0.01 | 0.02 | 0.01 | 0.01 |
|  | [0.01 - 0.01] | [0.01 - 0.03] | [0.01 - 0.01] | [0.01 - 0.01] |
| Ethnicity = South Asian | 0.02 | 0.02 | 0.02 | 0.01 |
|  | [0.02 - 0.02] | [0.01 - 0.02] | [0.01 - 0.03] | [0.01 - 0.01] |
| Ethnicity = Black | 0.01 | 0.04 | 0.02 | 0.01 |
|  | [0.01 - 0.01] | [0.03 - 0.06] | [0.01 - 0.03] | [0.01 - 0.01] |
| Ethnicity = Other | 0.01 | 0.02 | 0.01 | 0.01 |
|  | [0.01 - 0.01] | [0.01 - 0.03] | [0.01 - 0.01] | [0.01 - 0.01] |
| Rural = Urban | 0.85 | 0.93 | 0.90 | 0.86 |
|  | [0.84 - 0.85] | [0.91 - 0.94] | [0.88 - 0.91] | [0.86 - 0.86] |
| Rural = Rural | 0.15 | 0.07 | 0.10 | 0.14 |
|  | [0.15 - 0.16] | [0.06 - 0.09] | [0.09 - 0.12] | [0.14 - 0.14] |
| **Socioeconomic:** Education = College or University | 0.38 | 0.30 | 0.40 | 0.34 |
|  | [0.38 - 0.38] | [0.27 - 0.32] | [0.38 - 0.42] | [0.33 - 0.34] |
| Education = A/AS levels | 0.12 | 0.16 | 0.13 | 0.12 |
|  | [0.12 - 0.12] | [0.14 - 0.18] | [0.12 - 0.15] | [0.12 - 0.12] |
| Education = O levels / GCSE | 0.26 | 0.24 | 0.25 | 0.28 |
|  | [0.26 - 0.27] | [0.22 - 0.27] | [0.23 - 0.26] | [0.27 - 0.28] |
| Education = Other | 0.11 | 0.10 | 0.10 | 0.12 |
|  | [0.11 - 0.11] | [0.08 - 0.11] | [0.09 - 0.11] | [0.12 - 0.12] |
| Education = None of the above | 0.12 | 0.21 | 0.12 | 0.15 |
|  | [0.12-0.13] | [0.18-0.23] | [0.10-0.13] | [0.14-0.15] |
| Townsend deprivation index (mean)^a^ | -1.62 | 1.25 | -0.26 | -1.08 |
|  | [-1.63 - -1.6] | [1.03 - 1.46] | [-0.4 - -0.12] | [-1.1 - -1.06] |
| Employment Status = Paid employment | 0.62 | 0.26 | 0.48 | 0.56 |
|  | [0.62 - 0.62] | [0.23 - 0.28] | [0.46 - 0.5] | [0.56 - 0.57] |
| Employment Status = Retired | 0.32 | 0.32 | 0.30 | 0.31 |
|  | [0.32 - 0.32] | [0.29 - 0.35] | [0.28 - 0.32] | [0.31 - 0.32] |
| Employment Status = Looking after home/family | 0.02 | 0.03 | 0.03 | 0.03 |
|  | [0.02 - 0.02] | [0.02 - 0.04] | [0.02 - 0.03] | [0.03 - 0.03] |
| Employment Status = Unable to work | 0.01 | 0.32 | 0.14 | 0.06 |
|  | [0.01 - 0.01] | [0.29 - 0.35] | [0.13 - 0.15] | [0.06 - 0.07] |
| Employment Status = Unemployed | 0.01 | 0.04 | 0.03 | 0.02 |
|  | [0.01 - 0.01] | [0.03 - 0.05] | [0.02 - 0.04] | [0.02 - 0.02] |
| Employment Status = Other | 0.01 | 0.03 | 0.03 | 0.01 |
|  | [0.01 - 0.01] | [0.02 - 0.04] | [0.02 - 0.03] | [0.01 - 0.01] |
| Housing = Own | 0.93 | 0.55 | 0.77 | 0.86 |
|  | [0.93 - 0.93] | [0.52 - 0.58] | [0.75 - 0.79] | [0.85 - 0.86] |
| Housing = Rent/Other | 0.07 | 0.45 | 0.23 | 0.14 |
|  | [0.07 - 0.07] | [0.42 - 0.48] | [0.21 - 0.25] | [0.14 - 0.15] |
| Household Income: < £18,000 | 0.18 | 0.66 | 0.37 | 0.29 |
|  | [0.18 - 0.18] | [0.63 - 0.69] | [0.35 - 0.39] | [0.29 - 0.29] |
| Household Income: 18,000 – 30,999 | 0.25 | 0.19 | 0.24 | 0.26 |
|  | [0.25 - 0.25] | [0.16 - 0.21] | [0.22 - 0.26] | [0.25 - 0.26] |
| Household Income: 31,000 – 51,999 | 0.27 | 0.09 | 0.21 | 0.24 |
|  | [0.27 - 0.27] | [0.07 - 0.11] | [0.2 - 0.23] | [0.24 - 0.25] |
| Household Income: 52,000 – 100,000 | 0.23 | 0.05 | 0.14 | 0.17 |
|  | [0.23 - 0.23] | [0.04 - 0.06] | [0.13 - 0.16] | [0.17 - 0.18] |
| Household Income: > 100,000 | 0.07 | 0.02 | 0.03 | 0.04 |
|  | [0.07 - 0.07] | [0.01 - 0.03] | [0.03 - 0.04] | [0.04 - 0.04] |
| **Health & Biomarkers:** BMI = Underweight | 0.00 | 0.02 | 0.01 | 0.01 |
|  | [0 - 0] | [0.01 - 0.02] | [0 - 0.01] | [0.01 - 0.01] |
| BMI = Normal | 0.34 | 0.27 | 0.29 | 0.32 |
|  | [0.34 - 0.35] | [0.24 - 0.29] | [0.27 - 0.31] | [0.31 - 0.32] |
| BMI = Overweight | 0.43 | 0.35 | 0.38 | 0.40 |
|  | [0.43 - 0.44] | [0.33 - 0.38] | [0.36 - 0.4] | [0.4 - 0.41] |
| BMI = Obese | 0.22 | 0.36 | 0.32 | 0.27 |
|  | [0.21 - 0.22] | [0.33 - 0.39] | [0.3 - 0.34] | [0.27 - 0.28] |
| Pulse Rate (mean) | 68.86 | 75.85 | 70.66 | 70.07 |
|  | [68.81 - 68.91] | [75.01 - 76.69] | [70.12 - 71.2] | [69.98 - 70.16] |
| Waist circumference (mean) | 89.97 | 96.20 | 93.75 | 90.67 |
|  | [89.91 - 90.02] | [95.31 - 97.09] | [93.11 - 94.39] | [90.56 - 90.79] |
| C-reactive Protein [log] | 0.24 | 0.67 | 0.47 | 0.41 |
|  | [0.23 - 0.24] | [0.59 - 0.74] | [0.42 - 0.52] | [0.4 - 0.42] |
| Number of Comorbidities = 0 | 0.40 | 0.14 | 0.20 | 0.29 |
|  | [0.39 - 0.4] | [0.12 - 0.16] | [0.18 - 0.21] | [0.28 - 0.29] |
| Number of Comorbidities = 1 | 0.34 | 0.28 | 0.29 | 0.32 |
|  | [0.33 - 0.34] | [0.26 - 0.31] | [0.27 - 0.31] | [0.32 - 0.32] |
| Number of Comorbidities = 2 | 0.17 | 0.32 | 0.25 | 0.21 |
|  | [0.17 - 0.17] | [0.29 - 0.34] | [0.23 - 0.27] | [0.21 - 0.21] |
| Number of Comorbidities = 3 | 0.07 | 0.14 | 0.14 | 0.11 |
|  | [0.06 - 0.07] | [0.12 - 0.16] | [0.12 - 0.15] | [0.1 - 0.11] |
| Number of Comorbidities = 4+ | 0.03 | 0.12 | 0.12 | 0.08 |
|  | [0.03 - 0.03] | [0.1 - 0.14] | [0.1 - 0.13] | [0.07 - 0.08] |
| **Health-related behaviours:** Smoking = Never | 0.59 | 0.43 | 0.43 | 0.49 |
|  | [0.59 - 0.59] | [0.4 - 0.46] | [0.41 - 0.45] | [0.49 - 0.49] |
| Smoking = Previous | 0.35 | 0.29 | 0.35 | 0.37 |
|  | [0.35 - 0.36] | [0.26 - 0.31] | [0.33 - 0.37] | [0.37 - 0.37] |
| Smoking = Current | 0.06 | 0.29 | 0.22 | 0.14 |
|  | [0.06 - 0.06] | [0.26 - 0.31] | [0.2 - 0.24] | [0.14 - 0.14] |
| Alcohol = Daily or almost daily | 0.22 | 0.17 | 0.22 | 0.20 |
|  | [0.22 - 0.22] | [0.15 - 0.2] | [0.2 - 0.24] | [0.2 - 0.21] |
| Alcohol = 3-4 times/week | 0.26 | 0.14 | 0.17 | 0.21 |
|  | [0.25 - 0.26] | [0.12 - 0.16] | [0.15 - 0.18] | [0.21 - 0.22] |
| Alcohol = 1-2 times/week | 0.26 | 0.19 | 0.22 | 0.24 |
|  | [0.26 - 0.26] | [0.16 - 0.21] | [0.21 - 0.24] | [0.24 - 0.25] |
| Alcohol = 1-3 times/month | 0.11 | 0.11 | 0.12 | 0.12 |
|  | [0.1 - 0.11] | [0.09 - 0.13] | [0.11 - 0.13] | [0.12 - 0.12] |
| Alcohol = Special occasions only | 0.10 | 0.16 | 0.13 | 0.13 |
|  | [0.09 - 0.1] | [0.13 - 0.18] | [0.12 - 0.15] | [0.12 - 0.13] |
| Alcohol = Never (former drinker) | 0.03 | 0.18 | 0.08 | 0.05 |
|  | [0.03 - 0.03] | [0.15 - 0.2] | [0.07 - 0.09] | [0.05 - 0.06] |
| Alcohol = Never | 0.04 | 0.06 | 0.05 | 0.04 |
|  | [0.03 - 0.04] | [0.05 - 0.07] | [0.04 - 0.06] | [0.04 - 0.04] |
| Physical Activity Quintile = 1 | 0.19 | 0.31 | 0.22 | 0.22 |
|  | [0.19 - 0.19] | [0.28 - 0.34] | [0.2 - 0.23] | [0.22 - 0.22] |
| Physical Activity Quintile = 2 | 0.20 | 0.22 | 0.21 | 0.20 |
|  | [0.2 - 0.2] | [0.19 - 0.24] | [0.19 - 0.23] | [0.2 - 0.2] |
| Physical Activity Quintile = 3 | 0.20 | 0.18 | 0.19 | 0.19 |
|  | [0.2 - 0.21] | [0.15 - 0.2] | [0.17 - 0.2] | [0.19 - 0.19] |
| Physical Activity Quintile = 4 | 0.21 | 0.14 | 0.19 | 0.19 |
|  | [0.21 - 0.21] | [0.12 - 0.16] | [0.17 - 0.21] | [0.19 - 0.19] |
| Physical Activity Quintile = 5 | 0.20 | 0.16 | 0.20 | 0.20 |
|  | [0.2 - 0.2] | [0.14 - 0.18] | [0.18 - 0.22] | [0.2 - 0.2] |
| **Social isolation:** Household Structure = Live with spouse/partner | 0.77 | 0.35 | 0.58 | 0.65 |
|  | [0.76 - 0.77] | [0.32 - 0.38] | [0.56 - 0.6] | [0.65 - 0.65] |
| Household Structure = Live with other person | 0.07 | 0.10 | 0.13 | 0.11 |
|  | [0.07 - 0.07] | [0.09 - 0.12] | [0.11 - 0.14] | [0.1 - 0.11] |
| Household Structure = Live alone | 0.17 | 0.55 | 0.29 | 0.24 |
|  | [0.17 - 0.17] | [0.52 - 0.58] | [0.27 - 0.31] | [0.24 - 0.25] |
| Visits friends/family ≥ 1/week | 0.78 | 0.70 | 0.75 | 0.78 |
|  | [0.78 - 0.78] | [0.67 - 0.72] | [0.73 - 0.77] | [0.78 - 0.79] |
| Visits friends/family < 1/week | 0.22 | 0.30 | 0.25 | 0.22 |
|  | [0.22 - 0.22] | [0.28 - 0.33] | [0.23 - 0.27] | [0.21 - 0.22] |
| Leisure/social activities ≥ 1/week | 0.72 | 0.69 | 0.71 | 0.68 |
|  | [0.72 - 0.72] | [0.66 - 0.72] | [0.69 - 0.73] | [0.68 - 0.69] |
| Leisure/social activities < 1/week | 0.28 | 0.31 | 0.29 | 0.32 |
|  | [0.28 - 0.28] | [0.28 - 0.34] | [0.27 - 0.31] | [0.31 - 0.32] |
| **Psychological factors:** Lonely = No | 0.87 | 0.60 | 0.59 | 0.69 |
|  | [0.87 - 0.87] | [0.57 - 0.63] | [0.57 - 0.61] | [0.69 - 0.69] |
| Lonely = Yes | 0.13 | 0.40 | 0.41 | 0.31 |
|  | [0.13 - 0.13] | [0.37 - 0.43] | [0.39 - 0.43] | [0.31 - 0.31] |
| PHQ (mean) | 1.23 | 3.59 | 3.42 | 2.61 |
|  | [1.22 - 1.23] | [3.39 - 3.78] | [3.28 - 3.55] | [2.59 - 2.63] |
| Insomnia = Never/rarely | 0.28 | 0.20 | 0.19 | 0.18 |
|  | [0.28 - 0.28] | [0.18 - 0.23] | [0.18 - 0.21] | [0.18 - 0.19] |
| Insomnia = Sometimes | 0.48 | 0.48 | 0.44 | 0.45 |
|  | [0.48 - 0.48] | [0.45 - 0.51] | [0.42 - 0.46] | [0.45 - 0.46] |
| Insomnia = Usually | 0.24 | 0.32 | 0.37 | 0.36 |
|  | [0.24 - 0.25] | [0.29 - 0.35] | [0.35 - 0.39] | [0.36 - 0.37] |

95% confidence intervals in brackets

^a^ Z score (higher values reflect greater deprivation)

Table S4: Full results from the Cox Proportional Hazard models (first admission) for the association between SCMD and ACSC admissions

|  | **M1** |  |  | **M2** |  |  | **M3** |  |  | **M4** |  |  | **M5** |  |  | **M6** |  |  | **M7** |  |  | **M8** |  |  |
| --- | --- | --- | --- | --- | --- | --- | --- | --- | --- | --- | --- | --- | --- | --- | --- | --- | --- | --- | --- | --- | --- | --- | --- | --- |
|  | **HR** | **L** | **U** | **HR** | **L** | **U** | **HR** | **L** | **U** | **HR** | **L** | **U** | **HR** | **L** | **U** | **HR** | **L** | **U** | **HR** | **L** | **U** | **HR** | **L** | **U** |
| **No SCMD** | 1.00 |  |  | 1.00 |  |  | 1.00 |  |  | 1.00 |  |  | 1.00 |  |  | 1.00 |  |  | 1.00 |  |  | 1.00 |  |  |
| Schizophrenia | 4.40 | 4.04 | 4.80 | 4.86 | 4.44 | 5.32 | 2.65 | 2.37 | 2.97 | 2.31 | 2.05 | 2.61 | 2.47 | 2.19 | 2.79 | 2.65 | 2.36 | 2.97 | 2.56 | 2.26 | 2.89 | 2.15 | 1.87 | 2.48 |
| Bipolar disorder | 2.48 | 2.28 | 2.69 | 2.81 | 2.58 | 3.06 | 2.12 | 1.92 | 2.33 | 1.72 | 1.55 | 1.90 | 1.95 | 1.76 | 2.16 | 2.10 | 1.90 | 2.31 | 1.95 | 1.76 | 2.16 | 1.56 | 1.39 | 1.76 |
| Anxiety or depression | 1.76 | 1.73 | 1.80 | 1.98 | 1.93 | 2.02 | 1.72 | 1.68 | 1.77 | 1.53 | 1.49 | 1.57 | 1.63 | 1.59 | 1.67 | 1.71 | 1.67 | 1.75 | 1.59 | 1.55 | 1.64 | 1.43 | 1.39 | 1.47 |
| Age (continuous) |  |  |  | 1.07 | 1.07 | 1.07 | 1.06 | 1.06 | 1.06 | 1.05 | 1.05 | 1.05 | 1.06 | 1.06 | 1.06 | 1.06 | 1.06 | 1.06 | 1.06 | 1.06 | 1.06 | 1.05 | 1.05 | 1.05 |
| **Sex = Female** |  |  |  | 1.00 |  |  | 1.00 |  |  | 1.00 |  |  | 1.00 |  |  | 1.00 |  |  | 1.00 |  |  | 1.00 |  |  |
| Sex = Male |  |  |  | 1.40 | 1.37 | 1.42 | 1.39 | 1.36 | 1.42 | 1.30 | 1.26 | 1.34 | 1.44 | 1.41 | 1.47 | 1.39 | 1.36 | 1.43 | 1.41 | 1.38 | 1.45 | 1.34 | 1.30 | 1.38 |
| **Ethnicity = White British** |  |  |  | 1.00 |  |  | 1.00 |  |  | 1.00 |  |  | 1.00 |  |  | 1.00 |  |  | 1.00 |  |  | 1.00 |  |  |
| Ethnicity = White Irish |  |  |  | 1.11 | 1.05 | 1.18 | 1.05 | 0.98 | 1.12 | 1.05 | 0.98 | 1.12 | 1.02 | 0.96 | 1.10 | 1.05 | 0.98 | 1.12 | 1.06 | 0.99 | 1.13 | 1.03 | 0.96 | 1.11 |
| Ethnicity = White Other |  |  |  | 0.93 | 0.88 | 0.99 | 0.95 | 0.89 | 1.01 | 0.96 | 0.89 | 1.03 | 0.93 | 0.86 | 0.99 | 0.94 | 0.88 | 1.01 | 0.93 | 0.86 | 0.99 | 0.93 | 0.86 | 1.00 |
| Ethnicity = Mixed |  |  |  | 1.14 | 1.00 | 1.31 | 1.02 | 0.87 | 1.19 | 1.06 | 0.91 | 1.25 | 0.99 | 0.84 | 1.16 | 1.02 | 0.87 | 1.19 | 1.04 | 0.88 | 1.22 | 1.06 | 0.89 | 1.26 |
| Ethnicity = South Asian |  |  |  | 1.39 | 1.30 | 1.48 | 1.18 | 1.09 | 1.28 | 1.09 | 1.00 | 1.19 | 1.07 | 0.98 | 1.17 | 1.19 | 1.10 | 1.29 | 1.10 | 1.00 | 1.21 | 1.02 | 0.91 | 1.13 |
| Ethnicity = Black |  |  |  | 1.38 | 1.28 | 1.49 | 1.07 | 0.97 | 1.17 | 1.07 | 0.97 | 1.18 | 1.06 | 0.96 | 1.16 | 1.08 | 0.98 | 1.18 | 1.07 | 0.97 | 1.19 | 1.07 | 0.96 | 1.20 |
| Ethnicity = Other |  |  |  | 1.20 | 1.10 | 1.31 | 1.03 | 0.92 | 1.15 | 1.05 | 0.93 | 1.18 | 0.96 | 0.85 | 1.07 | 1.04 | 0.93 | 1.16 | 0.98 | 0.87 | 1.11 | 0.95 | 0.83 | 1.10 |
| **Rural = Urban** |  |  |  | 1.00 |  |  | 1.00 |  |  | 1.00 |  |  | 1.00 |  |  | 1.00 |  |  | 1.00 |  |  | 1.00 |  |  |
| Rural = Rural |  |  |  | 0.84 | 0.82 | 0.87 | 0.95 | 0.92 | 0.98 | 0.96 | 0.93 | 0.99 | 0.96 | 0.93 | 0.99 | 0.95 | 0.92 | 0.98 | 0.96 | 0.93 | 0.99 | 0.98 | 0.94 | 1.01 |
| **Education = College or University** |  |  |  |  |  |  | 1.00 |  |  | 1.00 |  |  | 1.00 |  |  | 1.00 |  |  | 1.00 |  |  | 1.00 |  |  |
| Education = A/AS levels |  |  |  |  |  |  | 1.04 | 1.00 | 1.08 | 1.00 | 0.96 | 1.05 | 1.03 | 0.98 | 1.07 | 1.04 | 1.00 | 1.08 | 1.04 | 0.99 | 1.08 | 0.99 | 0.95 | 1.04 |
| Education = O levels / GCSE |  |  |  |  |  |  | 1.11 | 1.08 | 1.15 | 1.07 | 1.03 | 1.10 | 1.09 | 1.06 | 1.12 | 1.11 | 1.08 | 1.15 | 1.10 | 1.06 | 1.13 | 1.04 | 1.00 | 1.08 |
| Education = Other |  |  |  |  |  |  | 1.17 | 1.13 | 1.21 | 1.09 | 1.05 | 1.14 | 1.13 | 1.09 | 1.17 | 1.17 | 1.12 | 1.21 | 1.16 | 1.12 | 1.20 | 1.07 | 1.03 | 1.12 |
| Education = None of the above |  |  |  |  |  |  | 1.26 | 1.22 | 1.30 | 1.17 | 1.13 | 1.21 | 1.19 | 1.15 | 1.23 | 1.25 | 1.21 | 1.29 | 1.23 | 1.18 | 1.27 | 1.12 | 1.07 | 1.16 |
| Townsend deprivation index (continuous) |  |  |  |  |  |  | 1.03 | 1.02 | 1.03 | 1.01 | 1.01 | 1.02 | 1.02 | 1.02 | 1.03 | 1.03 | 1.02 | 1.03 | 1.02 | 1.02 | 1.03 | 1.01 | 1.00 | 1.01 |
| **Employment Status = Paid employment** |  |  |  |  |  |  | 1.00 |  |  | 1.00 |  |  | 1.00 |  |  | 1.00 |  |  | 1.00 |  |  | 1.00 |  |  |
| Employment Status = Retired |  |  |  |  |  |  | 1.13 | 1.10 | 1.17 | 1.06 | 1.03 | 1.09 | 1.13 | 1.09 | 1.16 | 1.15 | 1.12 | 1.18 | 1.12 | 1.08 | 1.15 | 1.05 | 1.02 | 1.09 |
| Employment Status = Looking after home/family |  |  |  |  |  |  | 1.07 | 0.99 | 1.17 | 1.01 | 0.92 | 1.10 | 1.05 | 0.96 | 1.14 | 1.08 | 1.00 | 1.18 | 1.04 | 0.95 | 1.14 | 0.98 | 0.89 | 1.09 |
| Employment Status = Unable to work |  |  |  |  |  |  | 2.24 | 2.13 | 2.35 | 1.52 | 1.45 | 1.61 | 1.94 | 1.83 | 2.05 | 2.24 | 2.13 | 2.36 | 1.97 | 1.86 | 2.08 | 1.39 | 1.31 | 1.49 |
| Employment Status = Unemployed |  |  |  |  |  |  | 1.10 | 1.01 | 1.20 | 1.04 | 0.95 | 1.14 | 1.08 | 0.99 | 1.19 | 1.11 | 1.01 | 1.21 | 1.08 | 0.98 | 1.18 | 1.00 | 0.90 | 1.12 |
| Employment Status = Other |  |  |  |  |  |  | 1.09 | 0.98 | 1.21 | 1.06 | 0.95 | 1.19 | 1.10 | 0.98 | 1.22 | 1.10 | 0.99 | 1.22 | 1.04 | 0.93 | 1.17 | 1.02 | 0.90 | 1.16 |
| **Housing = Own** |  |  |  |  |  |  | 1.00 |  |  | 1.00 |  |  | 1.00 |  |  | 1.00 |  |  | 1.00 |  |  | 1.00 |  |  |
| Housing = Rent/Other |  |  |  |  |  |  | 1.31 | 1.26 | 1.35 | 1.19 | 1.14 | 1.23 | 1.20 | 1.16 | 1.25 | 1.29 | 1.24 | 1.33 | 1.27 | 1.23 | 1.32 | 1.09 | 1.05 | 1.14 |
| **Household Income: < £18,000** |  |  |  |  |  |  | 1.00 |  |  | 1.00 |  |  | 1.00 |  |  | 1.00 |  |  | 1.00 |  |  | 1.00 |  |  |
| HH Income: 18,000 – 30,999 |  |  |  |  |  |  | 0.89 | 0.86 | 0.91 | 0.91 | 0.88 | 0.94 | 0.91 | 0.88 | 0.94 | 0.90 | 0.87 | 0.93 | 0.90 | 0.87 | 0.93 | 0.93 | 0.90 | 0.96 |
| HH Income: 31,000 – 51,999 |  |  |  |  |  |  | 0.83 | 0.80 | 0.86 | 0.86 | 0.83 | 0.89 | 0.86 | 0.83 | 0.89 | 0.84 | 0.81 | 0.87 | 0.84 | 0.81 | 0.87 | 0.89 | 0.85 | 0.93 |
| HH Income: 52,000 – 100,000 |  |  |  |  |  |  | 0.76 | 0.73 | 0.79 | 0.80 | 0.76 | 0.83 | 0.80 | 0.76 | 0.83 | 0.77 | 0.74 | 0.81 | 0.77 | 0.73 | 0.80 | 0.83 | 0.79 | 0.88 |
| HH Income: > 100,000 |  |  |  |  |  |  | 0.71 | 0.66 | 0.76 | 0.76 | 0.70 | 0.81 | 0.76 | 0.71 | 0.82 | 0.73 | 0.68 | 0.78 | 0.73 | 0.68 | 0.78 | 0.80 | 0.74 | 0.86 |
| **Number of Comorbidities = 0** |  |  |  |  |  |  |  |  |  | 1.00 |  |  |  |  |  |  |  |  |  |  |  | 1.00 |  |  |
| Number of Comorbidities = 1 |  |  |  |  |  |  |  |  |  | 1.38 | 1.34 | 1.43 |  |  |  |  |  |  |  |  |  | 1.36 | 1.32 | 1.41 |
| Number of Comorbidities = 2 |  |  |  |  |  |  |  |  |  | 1.76 | 1.70 | 1.82 |  |  |  |  |  |  |  |  |  | 1.71 | 1.65 | 1.78 |
| Number of Comorbidities = 3 |  |  |  |  |  |  |  |  |  | 2.33 | 2.24 | 2.43 |  |  |  |  |  |  |  |  |  | 2.23 | 2.13 | 2.33 |
| Number of Comorbidities = 4+ |  |  |  |  |  |  |  |  |  | 3.15 | 3.01 | 3.29 |  |  |  |  |  |  |  |  |  | 2.89 | 2.75 | 3.05 |
| BMI = Underweight |  |  |  |  |  |  |  |  |  | 2.19 | 1.90 | 2.53 |  |  |  |  |  |  |  |  |  | 2.02 | 1.72 | 2.38 |
| **BMI = Normal** |  |  |  |  |  |  |  |  |  | 1.00 |  |  |  |  |  |  |  |  |  |  |  | 1.00 |  |  |
| BMI = Overweight |  |  |  |  |  |  |  |  |  | 0.84 | 0.81 | 0.87 |  |  |  |  |  |  |  |  |  | 0.86 | 0.83 | 0.90 |
| BMI = Obese |  |  |  |  |  |  |  |  |  | 0.80 | 0.76 | 0.84 |  |  |  |  |  |  |  |  |  | 0.84 | 0.80 | 0.89 |
| Pulse (continuous) |  |  |  |  |  |  |  |  |  | 1.01 | 1.01 | 1.01 |  |  |  |  |  |  |  |  |  | 1.01 | 1.01 | 1.01 |
| Waist circumference (continuous) |  |  |  |  |  |  |  |  |  | 1.01 | 1.01 | 1.01 |  |  |  |  |  |  |  |  |  | 1.01 | 1.01 | 1.01 |
| C-reactive Protein [log] |  |  |  |  |  |  |  |  |  | 1.16 | 1.15 | 1.18 |  |  |  |  |  |  |  |  |  | 1.15 | 1.13 | 1.16 |
| **Smoking = Never** |  |  |  |  |  |  |  |  |  |  |  |  | 1.00 |  |  |  |  |  |  |  |  | 1.00 |  |  |
| Smoking = Previous |  |  |  |  |  |  |  |  |  |  |  |  | 1.24 | 1.21 | 1.27 |  |  |  |  |  |  | 1.15 | 1.12 | 1.18 |
| Smoking = Current |  |  |  |  |  |  |  |  |  |  |  |  | 1.53 | 1.47 | 1.59 |  |  |  |  |  |  | 1.47 | 1.41 | 1.54 |
| **Alcohol = Daily or almost daily** |  |  |  |  |  |  |  |  |  |  |  |  | 1.00 |  |  |  |  |  |  |  |  | 1.00 |  |  |
| Alcohol = 3-4 times/week |  |  |  |  |  |  |  |  |  |  |  |  | 0.95 | 0.91 | 0.98 |  |  |  |  |  |  | 0.96 | 0.92 | 1.00 |
| Alcohol = 1-2 times/week |  |  |  |  |  |  |  |  |  |  |  |  | 1.05 | 1.02 | 1.09 |  |  |  |  |  |  | 1.03 | 0.99 | 1.07 |
| Alcohol = 1-3 times/month |  |  |  |  |  |  |  |  |  |  |  |  | 1.12 | 1.08 | 1.17 |  |  |  |  |  |  | 1.06 | 1.01 | 1.11 |
| Alcohol = Special occasions only |  |  |  |  |  |  |  |  |  |  |  |  | 1.35 | 1.30 | 1.41 |  |  |  |  |  |  | 1.21 | 1.15 | 1.26 |
| Alcohol = Never (former drinker) |  |  |  |  |  |  |  |  |  |  |  |  | 1.51 | 1.43 | 1.59 |  |  |  |  |  |  | 1.34 | 1.26 | 1.42 |
| Alcohol = Never |  |  |  |  |  |  |  |  |  |  |  |  | 1.41 | 1.33 | 1.50 |  |  |  |  |  |  | 1.30 | 1.22 | 1.39 |
| **Physical Activity = 1** |  |  |  |  |  |  |  |  |  |  |  |  | 1.00 |  |  |  |  |  |  |  |  | 1.00 |  |  |
| Physical Activity = 2 |  |  |  |  |  |  |  |  |  |  |  |  | 0.90 | 0.87 | 0.93 |  |  |  |  |  |  | 0.96 | 0.93 | 1.00 |
| Physical Activity = 3 |  |  |  |  |  |  |  |  |  |  |  |  | 0.82 | 0.79 | 0.84 |  |  |  |  |  |  | 0.91 | 0.88 | 0.95 |
| Physical Activity = 4 |  |  |  |  |  |  |  |  |  |  |  |  | 0.82 | 0.79 | 0.85 |  |  |  |  |  |  | 0.95 | 0.92 | 0.99 |
| Physical Activity = 5 |  |  |  |  |  |  |  |  |  |  |  |  | 0.82 | 0.79 | 0.85 |  |  |  |  |  |  | 0.97 | 0.94 | 1.01 |
| **Household Structure = Living with spouse/partner** |  |  |  |  |  |  |  |  |  |  |  |  |  |  |  | 1.00 |  |  |  |  |  | 1.00 |  |  |
| Household Structure = Live with other person |  |  |  |  |  |  |  |  |  |  |  |  |  |  |  | 1.07 | 1.02 | 1.11 |  |  |  | 1.04 | 0.99 | 1.10 |
| Household Structure = Live alone |  |  |  |  |  |  |  |  |  |  |  |  |  |  |  | 1.04 | 1.01 | 1.07 |  |  |  | 1.04 | 1.01 | 1.08 |
| **Visits friends/family ≥ 1/week** |  |  |  |  |  |  |  |  |  |  |  |  |  |  |  | 1.00 |  |  |  |  |  | 1.00 |  |  |
| Visits friends/family < 1/week |  |  |  |  |  |  |  |  |  |  |  |  |  |  |  | 1.03 | 1.01 | 1.06 |  |  |  | 1.04 | 1.01 | 1.07 |
| **Leisure/social activities ≥ 1/week** |  |  |  |  |  |  |  |  |  |  |  |  |  |  |  | 1.00 |  |  |  |  |  | 1.00 |  |  |
| Leisure/social activities < 1/week |  |  |  |  |  |  |  |  |  |  |  |  |  |  |  | 1.13 | 1.10 | 1.16 |  |  |  | 0.99 | 0.97 | 1.02 |
| **Lonely = No** |  |  |  |  |  |  |  |  |  |  |  |  |  |  |  |  |  |  | 1.00 |  |  | 1.00 |  |  |
| Lonely = Yes |  |  |  |  |  |  |  |  |  |  |  |  |  |  |  |  |  |  | 1.00 | 0.97 | 1.03 | 0.99 | 0.95 | 1.02 |
| PHQ (continuous) |  |  |  |  |  |  |  |  |  |  |  |  |  |  |  |  |  |  | 1.05 | 1.05 | 1.06 | 1.02 | 1.01 | 1.03 |
| **Insomnia = Never/rarely** |  |  |  |  |  |  |  |  |  |  |  |  |  |  |  |  |  |  | 1.00 |  |  | 1.00 |  |  |
| Insomnia = Sometimes |  |  |  |  |  |  |  |  |  |  |  |  |  |  |  |  |  |  | 1.01 | 0.98 | 1.04 | 1.00 | 0.97 | 1.03 |
| Insomnia = Usually |  |  |  |  |  |  |  |  |  |  |  |  |  |  |  |  |  |  | 1.11 | 1.07 | 1.14 | 1.04 | 1.01 | 1.08 |

ACSC=Ambulatory Care Sensitive Conditions; BMI=Body Mass Index; HH=Household; HR=Hazard ratio; L=Lower 95% confidence interval; M=Model; PHQ=Patient Health Questionnaire; SCMD=Severe and common mental disorders; U=Upper 95% confidence interval; Reference categories are highlighted in bold

Table S5: Full results from the Prentice, Williams and Peterson Total Time (PWP-TT] models (all admissions) for the association between SCMD and ACSC admissions

|  | **M1** |  |  | **M2** |  |  | **M3** |  |  | **M4** |  |  | **M5** |  |  | **M6** |  |  | **M7** |  |  | **M8** |  |  |
| --- | --- | --- | --- | --- | --- | --- | --- | --- | --- | --- | --- | --- | --- | --- | --- | --- | --- | --- | --- | --- | --- | --- | --- | --- |
|  | **HR** | **L** | **U** | **HR** | **L** | **U** | **HR** | **L** | **U** | **HR** | **L** | **U** | **HR** | **L** | **U** | **HR** | **L** | **U** | **HR** | **L** | **U** | **HR** | **L** | **U** |
| **No SCMD** | 1.00 |  |  | 1.00 |  |  | 1.00 |  |  | 1.00 |  |  | 1.00 |  |  | 1.00 |  |  | 1.00 |  |  | 1.00 |  |  |
| Schizophrenia | 2.29 | 2.08 | 2.52 | 2.64 | 2.38 | 2.93 | 2.09 | 1.84 | 2.38 | 1.96 | 1.70 | 2.25 | 1.92 | 1.66 | 2.23 | 2.10 | 1.85 | 2.39 | 2.11 | 1.83 | 2.42 | 1.86 | 1.57 | 2.20 |
| Bipolar disorder | 1.92 | 1.77 | 2.08 | 2.13 | 1.95 | 2.33 | 1.79 | 1.62 | 1.98 | 1.58 | 1.42 | 1.76 | 1.84 | 1.68 | 2.03 | 1.78 | 1.61 | 1.97 | 1.73 | 1.55 | 1.92 | 1.62 | 1.46 | 1.80 |
| Anxiety or depression | 1.57 | 1.54 | 1.60 | 1.69 | 1.66 | 1.73 | 1.54 | 1.50 | 1.58 | 1.42 | 1.38 | 1.46 | 1.48 | 1.44 | 1.52 | 1.53 | 1.49 | 1.57 | 1.47 | 1.43 | 1.51 | 1.36 | 1.32 | 1.40 |
| Age (continuous) |  |  |  | 1.05 | 1.05 | 1.05 | 1.05 | 1.04 | 1.05 | 1.04 | 1.04 | 1.04 | 1.05 | 1.05 | 1.05 | 1.05 | 1.05 | 1.05 | 1.05 | 1.05 | 1.05 | 1.04 | 1.04 | 1.05 |
| **Sex = Female** |  |  |  | 1.00 |  |  | 1.00 |  |  | 1.00 |  |  | 1.00 |  |  | 1.00 |  |  | 1.00 |  |  | 1.00 |  |  |
| Sex = Male |  |  |  | 1.30 | 1.27 | 1.32 | 1.29 | 1.27 | 1.32 | 1.25 | 1.21 | 1.29 | 1.34 | 1.31 | 1.37 | 1.30 | 1.27 | 1.33 | 1.31 | 1.28 | 1.35 | 1.27 | 1.23 | 1.32 |
| **Ethnicity = White British** |  |  |  | 1.00 |  |  | 1.00 |  |  | 1.00 |  |  | 1.00 |  |  | 1.00 |  |  | 1.00 |  |  | 1.00 |  |  |
| Ethnicity = White Irish |  |  |  | 1.08 | 1.01 | 1.15 | 1.03 | 0.96 | 1.11 | 1.01 | 0.93 | 1.10 | 1.02 | 0.94 | 1.10 | 1.03 | 0.96 | 1.11 | 1.02 | 0.95 | 1.11 | 1.00 | 0.91 | 1.09 |
| Ethnicity = White Other |  |  |  | 0.97 | 0.92 | 1.03 | 1.00 | 0.94 | 1.07 | 0.99 | 0.92 | 1.07 | 0.99 | 0.93 | 1.07 | 1.00 | 0.93 | 1.06 | 0.97 | 0.91 | 1.05 | 0.99 | 0.91 | 1.07 |
| Ethnicity = Mixed |  |  |  | 1.06 | 0.93 | 1.22 | 1.00 | 0.85 | 1.17 | 1.04 | 0.91 | 1.19 | 1.03 | 0.89 | 1.18 | 1.01 | 0.86 | 1.18 | 1.07 | 0.91 | 1.25 | 1.07 | 0.93 | 1.24 |
| Ethnicity = South Asian |  |  |  | 1.16 | 1.08 | 1.25 | 1.03 | 0.94 | 1.13 | 0.99 | 0.90 | 1.08 | 0.97 | 0.88 | 1.07 | 1.03 | 0.94 | 1.13 | 0.98 | 0.88 | 1.10 | 0.98 | 0.88 | 1.10 |
| Ethnicity = Black |  |  |  | 1.32 | 1.22 | 1.42 | 1.13 | 1.03 | 1.23 | 1.13 | 1.03 | 1.24 | 1.16 | 1.05 | 1.27 | 1.14 | 1.04 | 1.24 | 1.13 | 1.03 | 1.25 | 1.19 | 1.07 | 1.33 |
| Ethnicity = Other |  |  |  | 1.17 | 1.07 | 1.27 | 1.01 | 0.91 | 1.12 | 1.06 | 0.95 | 1.18 | 0.99 | 0.89 | 1.10 | 1.03 | 0.93 | 1.14 | 0.95 | 0.84 | 1.06 | 0.97 | 0.85 | 1.10 |
| **Rural = Urban** |  |  |  | 1.00 |  |  | 1.00 |  |  | 1.00 |  |  | 1.00 |  |  | 1.00 |  |  | 1.00 |  |  | 1.00 |  |  |
| Rural = Rural |  |  |  | 0.88 | 0.85 | 0.91 | 0.97 | 0.93 | 1.00 | 0.97 | 0.94 | 1.00 | 0.97 | 0.93 | 1.00 | 0.97 | 0.93 | 1.00 | 0.97 | 0.94 | 1.01 | 0.98 | 0.95 | 1.02 |
| **Education = College or University** |  |  |  |  |  |  | 1.00 |  |  | 1.00 |  |  | 1.00 |  |  | 1.00 |  |  | 1.00 |  |  | 1.00 |  |  |
| Education = A/AS levels |  |  |  |  |  |  | 1.06 | 1.02 | 1.10 | 1.03 | 0.98 | 1.07 | 1.05 | 1.01 | 1.09 | 1.06 | 1.02 | 1.10 | 1.06 | 1.01 | 1.10 | 1.01 | 0.97 | 1.06 |
| Education = O levels / GCSE |  |  |  |  |  |  | 1.10 | 1.07 | 1.14 | 1.06 | 1.03 | 1.10 | 1.08 | 1.05 | 1.12 | 1.10 | 1.07 | 1.14 | 1.09 | 1.05 | 1.12 | 1.04 | 1.00 | 1.07 |
| Education = Other |  |  |  |  |  |  | 1.14 | 1.10 | 1.18 | 1.08 | 1.04 | 1.13 | 1.10 | 1.06 | 1.14 | 1.14 | 1.10 | 1.18 | 1.13 | 1.08 | 1.17 | 1.04 | 0.99 | 1.08 |
| Education = None of the above |  |  |  |  |  |  | 1.19 | 1.15 | 1.23 | 1.14 | 1.10 | 1.18 | 1.14 | 1.10 | 1.18 | 1.17 | 1.13 | 1.22 | 1.17 | 1.12 | 1.21 | 1.09 | 1.05 | 1.14 |
| Townsend deprivation index (continuous) |  |  |  |  |  |  | 1.02 | 1.01 | 1.02 | 1.01 | 1.00 | 1.01 | 1.01 | 1.01 | 1.02 | 1.02 | 1.01 | 1.02 | 1.01 | 1.01 | 1.02 | 1.00 | 1.00 | 1.01 |
| **Employment Status = Paid employment** |  |  |  |  |  |  | 1.00 |  |  | 1.00 |  |  | 1.00 |  |  | 1.00 |  |  | 1.00 |  |  | 1.00 |  |  |
| Employment Status = Retired |  |  |  |  |  |  | 1.11 | 1.07 | 1.14 | 1.03 | 1.00 | 1.06 | 1.08 | 1.05 | 1.12 | 1.12 | 1.09 | 1.15 | 1.09 | 1.06 | 1.13 | 1.02 | 0.98 | 1.06 |
| Employment Status = Looking after home/family |  |  |  |  |  |  | 1.10 | 1.02 | 1.20 | 1.05 | 0.97 | 1.15 | 1.08 | 0.99 | 1.18 | 1.12 | 1.03 | 1.21 | 1.07 | 0.98 | 1.17 | 1.04 | 0.94 | 1.15 |
| Employment Status = Unable to work |  |  |  |  |  |  | 1.63 | 1.55 | 1.72 | 1.28 | 1.21 | 1.36 | 1.51 | 1.43 | 1.60 | 1.64 | 1.56 | 1.73 | 1.49 | 1.41 | 1.58 | 1.22 | 1.13 | 1.30 |
| Employment Status = Unemployed |  |  |  |  |  |  | 1.21 | 1.11 | 1.32 | 1.13 | 1.04 | 1.23 | 1.20 | 1.10 | 1.30 | 1.22 | 1.12 | 1.33 | 1.21 | 1.10 | 1.33 | 1.09 | 0.99 | 1.21 |
| Employment Status = Other |  |  |  |  |  |  | 1.19 | 1.07 | 1.32 | 1.12 | 1.00 | 1.26 | 1.18 | 1.05 | 1.33 | 1.20 | 1.08 | 1.33 | 1.15 | 1.02 | 1.29 | 1.06 | 0.92 | 1.22 |
| **Housing = Own** |  |  |  |  |  |  | 1.00 |  |  | 1.00 |  |  | 1.00 |  |  | 1.00 |  |  | 1.00 |  |  | 1.00 |  |  |
| Housing = Rent/Other |  |  |  |  |  |  | 1.15 | 1.10 | 1.19 | 1.07 | 1.03 | 1.11 | 1.09 | 1.05 | 1.13 | 1.13 | 1.08 | 1.17 | 1.13 | 1.08 | 1.18 | 0.99 | 0.94 | 1.03 |
| **Household Income: < £18,000** |  |  |  |  |  |  | 1.00 |  |  | 1.00 |  |  | 1.00 |  |  | 1.00 |  |  | 1.00 |  |  | 1.00 |  |  |
| HH Income: 18,000 – 30,999 |  |  |  |  |  |  | 0.92 | 0.90 | 0.95 | 0.93 | 0.90 | 0.96 | 0.94 | 0.91 | 0.97 | 0.93 | 0.90 | 0.96 | 0.94 | 0.91 | 0.97 | 0.95 | 0.91 | 0.98 |
| HH Income: 31,000 – 51,999 |  |  |  |  |  |  | 0.85 | 0.82 | 0.88 | 0.87 | 0.84 | 0.90 | 0.87 | 0.84 | 0.91 | 0.86 | 0.83 | 0.89 | 0.87 | 0.84 | 0.90 | 0.90 | 0.86 | 0.93 |
| HH Income: 52,000 – 100,000 |  |  |  |  |  |  | 0.76 | 0.73 | 0.79 | 0.79 | 0.75 | 0.83 | 0.80 | 0.76 | 0.83 | 0.78 | 0.74 | 0.81 | 0.77 | 0.74 | 0.81 | 0.83 | 0.79 | 0.87 |
| HH Income: > 100,000 |  |  |  |  |  |  | 0.68 | 0.63 | 0.73 | 0.72 | 0.67 | 0.78 | 0.72 | 0.67 | 0.78 | 0.69 | 0.65 | 0.75 | 0.70 | 0.65 | 0.75 | 0.77 | 0.71 | 0.83 |
| **Number of Comorbidities = 0** |  |  |  |  |  |  |  |  |  | 1.00 |  |  |  |  |  |  |  |  |  |  |  | 1.00 |  |  |
| Number of Comorbidities = 1 |  |  |  |  |  |  |  |  |  | 1.43 | 1.38 | 1.48 |  |  |  |  |  |  |  |  |  | 1.40 | 1.35 | 1.45 |
| Number of Comorbidities = 2 |  |  |  |  |  |  |  |  |  | 1.80 | 1.74 | 1.87 |  |  |  |  |  |  |  |  |  | 1.75 | 1.68 | 1.82 |
| Number of Comorbidities = 3 |  |  |  |  |  |  |  |  |  | 2.17 | 2.08 | 2.27 |  |  |  |  |  |  |  |  |  | 2.06 | 1.97 | 2.16 |
| Number of Comorbidities = 4+ |  |  |  |  |  |  |  |  |  | 2.31 | 2.19 | 2.43 |  |  |  |  |  |  |  |  |  | 2.18 | 2.06 | 2.31 |
| BMI = Underweight |  |  |  |  |  |  |  |  |  | 1.88 | 1.61 | 2.18 |  |  |  |  |  |  |  |  |  | 1.69 | 1.40 | 2.04 |
| **BMI = Normal** |  |  |  |  |  |  |  |  |  | 1.00 |  |  |  |  |  |  |  |  |  |  |  | 1.00 |  |  |
| BMI = Overweight |  |  |  |  |  |  |  |  |  | 0.89 | 0.86 | 0.92 |  |  |  |  |  |  |  |  |  | 0.91 | 0.88 | 0.94 |
| BMI = Obese |  |  |  |  |  |  |  |  |  | 0.86 | 0.82 | 0.90 |  |  |  |  |  |  |  |  |  | 0.90 | 0.85 | 0.95 |
| Pulse (continuous) |  |  |  |  |  |  |  |  |  | 1.01 | 1.00 | 1.01 |  |  |  |  |  |  |  |  |  | 1.01 | 1.00 | 1.01 |
| Waist circumference (continuous) |  |  |  |  |  |  |  |  |  | 1.01 | 1.01 | 1.01 |  |  |  |  |  |  |  |  |  | 1.01 | 1.01 | 1.01 |
| C-reactive Protein [log] |  |  |  |  |  |  |  |  |  | 1.12 | 1.11 | 1.13 |  |  |  |  |  |  |  |  |  | 1.11 | 1.09 | 1.12 |
| **Smoking = Never** |  |  |  |  |  |  |  |  |  |  |  |  | 1.00 |  |  |  |  |  |  |  |  | 1.00 |  |  |
| Smoking = Previous |  |  |  |  |  |  |  |  |  |  |  |  | 1.17 | 1.15 | 1.20 |  |  |  |  |  |  | 1.12 | 1.09 | 1.15 |
| Smoking = Current |  |  |  |  |  |  |  |  |  |  |  |  | 1.39 | 1.34 | 1.45 |  |  |  |  |  |  | 1.40 | 1.34 | 1.47 |
| **Alcohol = Daily or almost daily** |  |  |  |  |  |  |  |  |  |  |  |  | 1.00 |  |  |  |  |  |  |  |  | 1.00 |  |  |
| Alcohol = 3-4 times/week |  |  |  |  |  |  |  |  |  |  |  |  | 0.94 | 0.90 | 0.97 |  |  |  |  |  |  | 0.96 | 0.93 | 1.00 |
| Alcohol = 1-2 times/week |  |  |  |  |  |  |  |  |  |  |  |  | 1.04 | 1.00 | 1.07 |  |  |  |  |  |  | 1.04 | 1.00 | 1.08 |
| Alcohol = 1-3 times/month |  |  |  |  |  |  |  |  |  |  |  |  | 1.08 | 1.03 | 1.13 |  |  |  |  |  |  | 1.05 | 1.01 | 1.11 |
| Alcohol = Special occasions only |  |  |  |  |  |  |  |  |  |  |  |  | 1.24 | 1.19 | 1.29 |  |  |  |  |  |  | 1.14 | 1.09 | 1.20 |
| Alcohol = Never (former drinker) |  |  |  |  |  |  |  |  |  |  |  |  | 1.30 | 1.23 | 1.38 |  |  |  |  |  |  | 1.20 | 1.13 | 1.29 |
| Alcohol = Never |  |  |  |  |  |  |  |  |  |  |  |  | 1.31 | 1.23 | 1.39 |  |  |  |  |  |  | 1.24 | 1.16 | 1.33 |
| **Physical Activity = 1** |  |  |  |  |  |  |  |  |  |  |  |  | 1.00 |  |  |  |  |  |  |  |  | 1.00 |  |  |
| Physical Activity = 2 |  |  |  |  |  |  |  |  |  |  |  |  | 0.93 | 0.90 | 0.96 |  |  |  |  |  |  | 0.98 | 0.94 | 1.02 |
| Physical Activity = 3 |  |  |  |  |  |  |  |  |  |  |  |  | 0.86 | 0.83 | 0.89 |  |  |  |  |  |  | 0.94 | 0.90 | 0.98 |
| Physical Activity = 4 |  |  |  |  |  |  |  |  |  |  |  |  | 0.88 | 0.85 | 0.91 |  |  |  |  |  |  | 0.98 | 0.94 | 1.02 |
| Physical Activity = 5 |  |  |  |  |  |  |  |  |  |  |  |  | 0.89 | 0.86 | 0.93 |  |  |  |  |  |  | 1.02 | 0.98 | 1.06 |
| **Household Structure = Living with spouse/partner** |  |  |  |  |  |  |  |  |  |  |  |  |  |  |  | 1.00 |  |  |  |  |  | 1.00 |  |  |
| Household Structure = Live with other person |  |  |  |  |  |  |  |  |  |  |  |  |  |  |  | 1.08 | 1.03 | 1.12 |  |  |  | 1.06 | 1.01 | 1.12 |
| Household Structure = Live alone |  |  |  |  |  |  |  |  |  |  |  |  |  |  |  | 1.03 | 1.00 | 1.06 |  |  |  | 1.03 | 1.00 | 1.07 |
| **Visits friends/family ≥ 1/week** |  |  |  |  |  |  |  |  |  |  |  |  |  |  |  | 1.00 |  |  |  |  |  | 1.00 |  |  |
| Visits friends/family < 1/week |  |  |  |  |  |  |  |  |  |  |  |  |  |  |  | 1.04 | 1.01 | 1.07 |  |  |  | 1.05 | 1.02 | 1.08 |
| **Leisure/social activities ≥ 1/week** |  |  |  |  |  |  |  |  |  |  |  |  |  |  |  | 1.00 |  |  |  |  |  | 1.00 |  |  |
| Leisure/social activities < 1/week |  |  |  |  |  |  |  |  |  |  |  |  |  |  |  | 1.10 | 1.07 | 1.12 |  |  |  | 1.00 | 0.97 | 1.03 |
| **Lonely = No** |  |  |  |  |  |  |  |  |  |  |  |  |  |  |  |  |  |  | 1.00 |  |  | 1.00 |  |  |
| Lonely = Yes |  |  |  |  |  |  |  |  |  |  |  |  |  |  |  |  |  |  | 1.00 | 0.97 | 1.04 | 0.99 | 0.95 | 1.03 |
| PHQ (continuous) |  |  |  |  |  |  |  |  |  |  |  |  |  |  |  |  |  |  | 1.03 | 1.03 | 1.04 | 1.01 | 1.00 | 1.02 |
| **Insomnia = Never/rarely** |  |  |  |  |  |  |  |  |  |  |  |  |  |  |  |  |  |  | 1.00 |  |  | 1.00 |  |  |
| Insomnia = Sometimes |  |  |  |  |  |  |  |  |  |  |  |  |  |  |  |  |  |  | 1.00 | 0.97 | 1.03 | 0.99 | 0.95 | 1.02 |
| Insomnia = Usually |  |  |  |  |  |  |  |  |  |  |  |  |  |  |  |  |  |  | 1.06 | 1.03 | 1.10 | 1.01 | 0.97 | 1.05 |

ACSC=Ambulatory Care Sensitive conditions; BMI=Body Mass Index; HH=Household; HR=Hazard ratio; L=Lower 95% confidence interval; M=Model; PHQ=Patient Health Questionnaire; SCMD=Severe and common mental disorders; U=Upper 95% confidence interval; Reference categories are highlighted in bold
